# Continuous In-Situ Polymerization of Complex-Based Films for High-Performance Electrochromic Devices

**DOI:** 10.3390/molecules30051099

**Published:** 2025-02-27

**Authors:** Yang-Bo Liu, Hao-Tian Deng, Li-Yi Zhang, Jing-Hao Wei, Feng-Rong Dai, Zhong-Ning Chen

**Affiliations:** 1 College of Chemistry, Fuzhou University, Fuzhou 350108, China; liuyangbo@fjirsm.ac.cn (Y.-B.L.); denghaotian@fjirsm.ac.cn (H.-T.D.); 2 State Key Laboratory of Structural Chemistry, Fujian Institute of Research on the Structure of Matter, Chinese Academy of Sciences, Fuzhou 350108, China; zhangliyi@fjirsm.ac.cn (L.-Y.Z.); weijinghao@fjirsm.ac.cn (J.-H.W.); 3 Fujian College, University of Chinese Academy of Sciences, Fuzhou 350002, China

**Keywords:** electrochromism, in situ polymerization, electrochromic film, Schiff base condensation, electrochromic devices

## Abstract

Synthesis of uniform and stable electrochromic films on a conductive layer is one of the effective ways to construct high-performance electrochromic devices. The development of more convenient and feasible polymer film preparation technology is important and necessary. Herein, we demonstrated the development of a continuous in situ polymerization method to prepare electrochromic film on ITO glass through Schiff base condensation of a tetraamine Fe-based complex and organic di-/tri-aldehyde precursors. The electrochromic film was successfully coated on the surface of the ITO conductive layer and exhibited uniform morphology and excellent stability. Film **P1** exhibited two reversible redox processes allowing two steps of electrochromic processes, including the oxidation of Fe(II) to Fe(III) at 1.05 V and oxidation of triphenylamine moieties to cation radicals at 1.4 V, which induced three stable color states from initial yellow to orange red and blue. The utilization of the so-formed polymer film for the fabrication of electrochromic devices gave rise to excellent electrochromic performance of fast response time of 0.4−1.2 s and high coloration efficiencies of 241.5−352.9 cm^2^/C at 1.9 V (at 535 nm) and 2.5 V (at 755 nm). The present work provides a new feasible strategy for constructing polymer films for high-performance electrochromic devices.

## 1. Introduction

Electrochromic devices (ECDs) featuring color changes with the change of external voltage have attracted wide attention and exhibit promising applications in a range of areas, such as smart windows, anti-glare rearview mirrors, electronic tags, wearable electronic devices, and electronic displays [1,2,3,4,5,6,7,8,9]. Typically, ECDs adopt a sandwich configuration consisting of three functional layers of electrochromic materials, electrolyte, and ion storage materials [8,9,10,11,12,13]. Therefore, coating electrochromic materials onto the surface of conductive substrates is an important factor in obtaining high-performance ECDs [13,14].

The common methods for constructing electrochromic films include spin coating, spray coating, vapor deposition, electrochemical polymerization, and photopolymerization. Spray coating has been applied to deposit thin films on ITO glass [15,16,17,18]; however, it requires the materials to have certain solubility or dispersibility, and the operator to have much experience to obtain high-quality thin films. The vapor deposition technology requires specialized instruments with high cost, which is not suitable for actual industrial production [19]. Electrochemical polymerization is also widely used in the preparation of electrochromic thin films [20,21,22,23,24,25]. Although this method has the advantages of short experimental period and controllable thickness tuned by polymerization time, it generally requires tedious synthesis steps to incorporate the specifically electroactive groups such as vinyl, thiophene, pyrrole, aromatic amines, and diphenylamine groups [26,27,28]. Furthermore, electropolymerization might produce unpredictable side products and/or oligomers on the surface of the electrode. The preparation of thin films by photopolymerization mainly relies on the photosensitivity of acryloyl groups, which polymerize into films under ultraviolet light irradiation [29,30,31]. Although this type of copolymer film has good flexibility and conductivity, the fussy synthetic procedures limit its widespread application [31].

Recently, Schiff base-mediated polycondensation has been developed to construct electrochromic film on the basis of the spray coating technique [16]. However, this coating method only provided small droplets of polymers on the surface of the ITO, thus leading to the electrochromic characteristics of long response time of 6.4–9.6 s and low coloration efficiency of 136.5 cm^2^/C. Therefore, to obtain high-performance complex polymer-based electrochromic devices, it is necessary to develop a more reliable and convenient method to fabricate a uniform electrochromic complex film on a conducting substrate. Herein, we developed an easily operational method using the continuous in situ polymerization technique to prepare a uniform electrochromic film on the ITO substrate. The so-formed electrochromic film had a uniform morphology adhered tightly to the surface of the ITO. The thickness of the polymer film is finely tunable by controlling the concentration of the starting materials. The so-formed polymer film exhibited excellent redox properties and electrochromic behaviors with a very short switching time of 1.6–1.9 s. After fabricating into a solid-state electrochromic device, excellent electrochromic performance with the shortest response time of 0.4 s and the highest coloration efficiency of 352.9 cm^2^/C was achieved.

## 2. Results and Discussion

### 2.1. Continuous In Situ Polymerization of Electrochromic Film on an ITO Substrate

**Figure 1:**
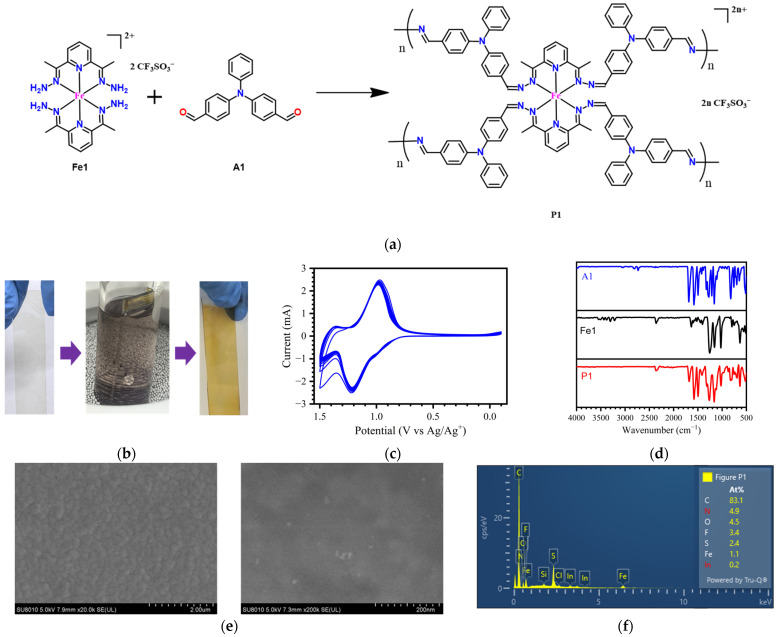
(**a**) Reaction scheme of **P1**; (**b**) images of the ITO glass before and after the continuous in situ polymerization; (**c**) cyclic voltammograms; (**d**) FT-IR spectra; (**e**) SEM images; and (**f**) EDS of electrochromic film **P1**.

The electrochromic films were conveniently grown on conductive ITO glass using a continuous in situ polymerization technique from a Fe-based complex (**Fe1**) [16] bearing tetraamine groups and organic 4,4′-diformyltriphenylamine (**A1**) (Figure 1a,b). ITO glass was placed into a chloroform solution of **Fe1** and **A1** (in a molar ratio of 1:2) containing a catalytic amount of trifluoroacetic acid. After heating up to 50 °C, the polycondensation of tetraamine and dialdehyde monomers occurred, and the polymer continuously grew on the ITO surface. After the reaction was complete, the ITO glass was taken out and ultrasonically cleaned with a triethylamine and acetonitrile solution to remove the trifluoroacetic acid and oligomers. Electrochromic film **P1** exhibited excellent stability and firmness on the surface of the ITO. Film **P1** showed excellent adhesion with the ITO, which did not peel off from the ITO surface even after several minutes of ultrasonic treatment in common organic solvents such as triethylamine, acetonitrile, ethanol, or dichloromethane. Moreover, no significant changes were observed after ten cycles of cyclic voltammetry scans (Figure 1c) in an acetonitrile solution of (Bu_4_N)PF_6_ (0.1 M). The CV curve demonstrated that film **P1** exhibited two stepwise oxidation/reduction processes. It underwent an oxidation process at 1.00 V involving the oxidation of Fe(II) to Fe(III) ions, and the second oxidation step, which occurred at 1.20 V, was attributed to the formation of radical cationic species by oxidizing the triphenylamine units. As depicted in Figure 1d, the absence of amine (–NH_2_) signals in the range of 3100–3600 cm^−1^ in the FT-IR spectrum of **P1** indicated the amine groups participated almost completely in the polymerization reaction as evidenced by the observation of the signal of C=N imine bonds at 1578 cm^−1^. As shown in Figure 1e, contrary to the polymer film generated using the on-substrate polymerization method [16], polymer **P1** covered the surface of the ITO, forming a continuous and uniform layer as revealed by scanning electron microscopy (SEM) (Figure 1b,c) and atomic force microscopy (AFM) (Figure S4) studies. Energy-dispersive X-ray spectroscopy (EDS) was conducted to further understand the elemental composition of the film (Figure 1f), which confirmed the presence of iron ions in the structure. These results clearly indicated the feasibility and superiority of the continuous in situ polymerization method for preparing electrochromic films.

**Figure 2:**
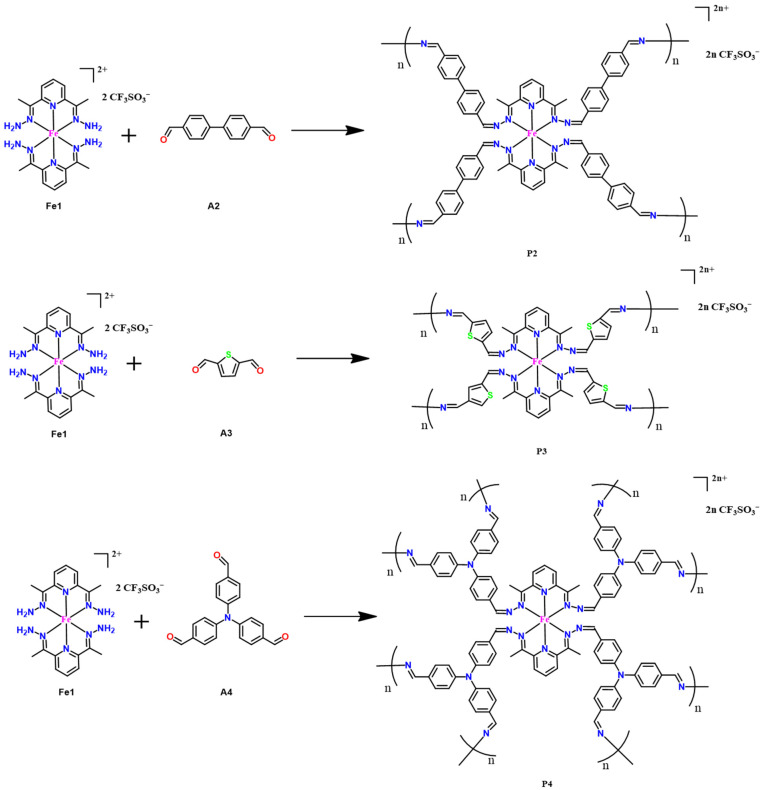
Reaction schemes of **P2**, **P3**, and **P4**.

To study the feasibility of varying the dialdehyde connector, linear dialdehyde of 4,4′-biphenyldicarboxaldehyde (**A2**), rigid angular dialdehyde of 2,5-thiophenedicarboxaldehyde (**A3**), and tri-aldehyde of tris(4-formylphenyl)amine (**A4**) were selected to react with Fe-based tetraamine **Fe1** under similar in situ polymerization conditions (Figure 2). The replacement of **A1** with **A2**, **A3**, or **A4** led to the formation of three new electrochromic films **P2**, **P3**, and **P4**, respectively. The FT-IR spectra demonstrated the complete conversion of amine (–NH_2_) units to imine (–C=N) species after reacting with the aldehyde groups (Figures S1–S3). The SEM and AFM analysis suggested that films **P2**–**P4** also had a smooth morphology and uniform coverage in the ITO (Figures S5–S10). Films **P2** (Figure S11) and **P3** (Figure S12) without the triphenylamine fragments underwent one oxidation/reduction process of the iron ions (Fe(II) ↔ Fe(III)) with the anodic and cathodic potentials of 1.50 and 0.60–0.75 V, respectively. Similarly to **P1**, film **P4** (Figure S13) bearing the triphenylamine groups showed two redox processes of the metal ions and the triphenylamine centers with oxidation potentials of 1.30 and 1.45 V, respectively. The successful in situ formation of films **P1**–**P4** films suggested the reliability of the in situ polymerization method and a great potential of extending to even more complex systems.

**Figure 3:**
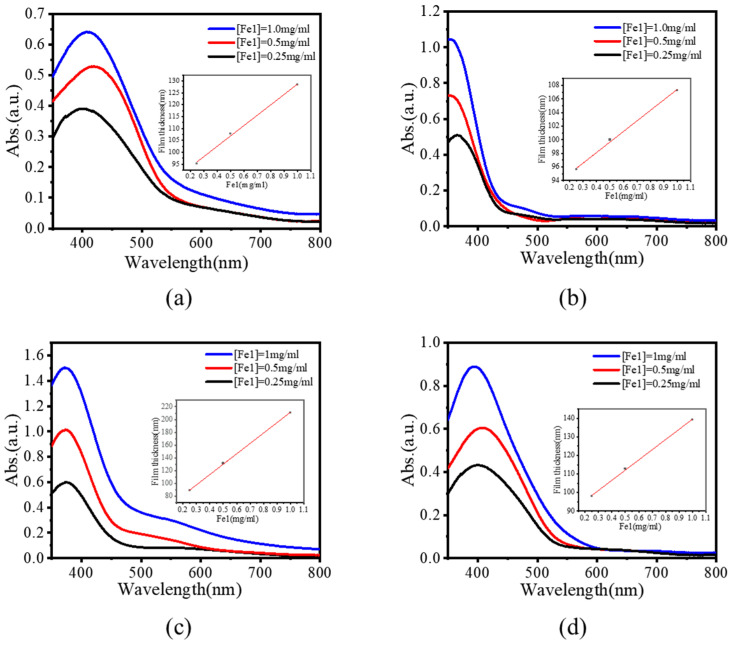
UV–vis absorption spectra of films (**a**) **P1**, (**b**) **P2**, (**c**) **P3**, and (**d**) **P4** on the ITO glass prepared from monomer solutions with various concentrations of **Fe1**.

Furthermore, we investigated the possibility of regulating film thickness by simply adjusting the concentration of monomer solutions. Figure 3 shows the UV–vis absorption spectra of films **P1**–**P4** prepared from different concentrations of tetraamine and di-/tri-aldehyde, with the concentration of **Fe1** changing from 0.25 to 1.00 mg/mL. It is worth noting that with the increase in [**Fe1**], the absorption intensities of the films increased correspondingly. The thicknesses of the films increased linearly with the increase in the concentration of reaction precursors (Figure 3). Moreover, as the thickness of the film changed, they still kept the same absorption bands that indicated the uniform deposition of the electrochromic films.

### 2.2. Spectroelectrochemical Properties of Electrochromic Films

**Figure 4:**
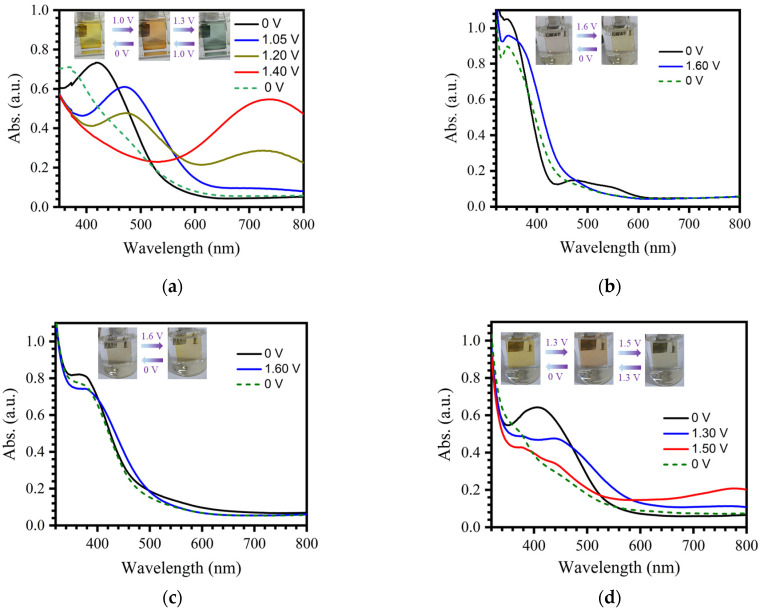
In situ UV–vis absorption spectra of electrochromic films (**a**) **P1**, (**b**) **P2**, (**c**) **P3**, and (**d**) **P4** under different external voltages. The insets show the color changes of electrochromic films after applying different external voltages.

The electrochromic properties of polymer films **P1**–**P4** were then evaluated through spectroelectrochemical studies. A three-electrode system was installed in a quartz cuvette using the electrochromic films (**P1**–**P4**) as the working electrode, a platinum plate as the counter electrode, and Ag/AgCl as the reference electrode. The in situ UV–vis absorption spectra of the electrochromic films after applying external voltages were measured using a combination of a UV–vis absorption spectrometer and an electrochemical workstation. As the applied voltage increased, the absorption band of **P1** centered at 420 nm gradually decreased, and a new absorption band appeared at 470 nm, which reached its maximum intensity at a driving voltage of 1.05 V (Figure 4a). At the same time, the color of film **P1** changed from yellow to orange-red. These phenomena can be attributed to the enhancement of the d–d transition and/or ligand-to-metal charge transfer (LMCT) induced by the oxidation of Fe(II) to Fe(III) under external voltage. When the driving voltage further increased, the absorption peak at 470 nm began to fall, and a new broad and intense absorption band centered at 745 nm raised quickly. It reached the steady state at the external potential of 1.40 V, accompanied by the change in the color of film **P1** to blue. This is ascribed to the oxidation of the triphenylamine fragments that further enhanced the LMCT between the triphenylamine radicals and Fe(III). It is worth noting that the colored states of film **P1** can be reversibly recovered to the original state after applying a potential bias of 0 V or under open-circuit conditions. Therefore, the electrochromic processes of film **P1** successfully exhibit three stable states under the external voltages of 0, 1.05, and 1.2 V, with color changes between yellow, orange-red, and blue, respectively.

When the applied external potential increased from 0 to 1.60 V during cyclic voltammetry at a scan rate of 5 mV/s, the UV–vis absorption of film **P2** displayed decreases in absorption intensities in the ranges of 300–365 and 470–600 nm and enhancement of the absorption intensity in the range of 365–470 nm (Figure 4b), indicating the successful oxidation of Fe(II) to Fe(III). During this process, the color of **P2** changed from pink to yellow. Film **P3** exhibited a similar electrochromic process as **P2**, with the color changed from gray to yellow (Figure 4c). Similarly to **P1**, film **P4,** constructed from triphenylamine-based tri-aldehyde, has two steps of electrochromic processes. As the applied voltage of the device gradually increased from 0 V to 1.3 V, the absorption band centered at 406 nm gradually decreased and red-shifted to 440 nm (Figure 4d), attributed to the oxidation of iron ions. As a result, the color of the film changed from yellow to orange-red. During the process of continuously increasing the applied voltage to 1.5 V, the absorption peaks in the range of 360–500 nm decreased and a broad absorption band centered at 770 nm appeared, resulting in the appearance of a blue color of the film. It should be noted that films **P2**–**P4** cannot fully recover to their initial state under an external voltage of 0 V, which might be due to the quasi-reversible oxidation/reduction properties of films **P2**–**P4** (Figures S14–S16).

**Figure 5:**
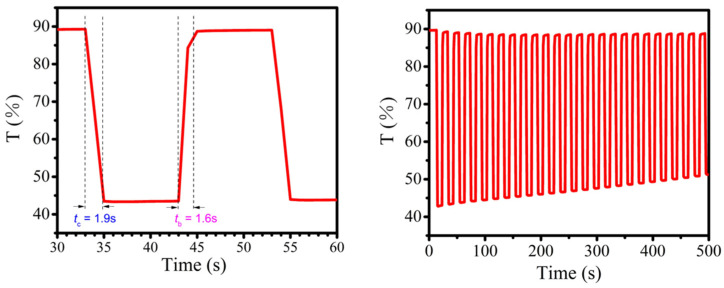
Electrochromic (**left**) switching times and (**right**) stability of the film at 745 nm.

The electrochemical stability and color switching times of film **P1** were further evaluated at stepping potentials between −0.1 and 1.2 V with an interval of 10 s monitoring by the transmittance at 745 nm, as shown in Figure 5. Film **P1** quickly switched from the initial state (yellow) to the oxidized state (blue) with a coloration time (*t*_c_) of 1.9 s after applying a potential of 1.2 V, and could reversibly return to the initial state with a slightly shorter bleaching time (*t*_b_) of 1.6 s. Meanwhile, an optical transmittance change (Δ*T*) of 45% was achieved. Film **P1** showed relative stability during 25 switching cycles, with the optical contrast dropping to 38%. Therefore, the fast response times and high optical contrast promoted **P1** as a promising electrochromic material layer for highly efficient electrochromic devices.

### 2.3. Fabrication and Characterization of Electrochromic Devices

**Figure 6:**
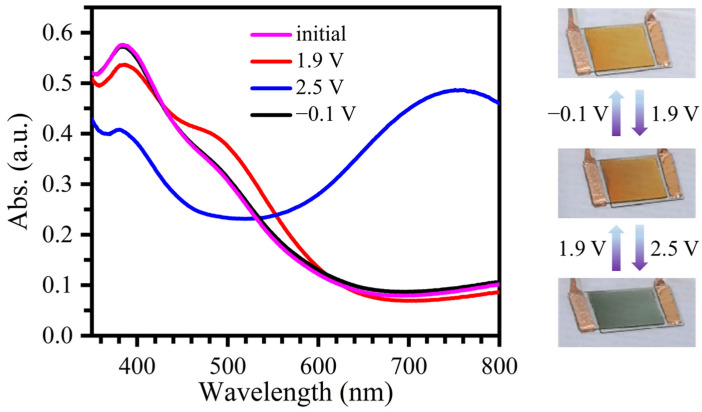
Photographs and in situ UV–vis absorption spectra of the electrochromic device based on film **P1** under different external voltages.

Electrochromic devices were finally fabricated in a sandwich structure of ITO/electrochromic film/gel electrolyte/TiO_2_/ITO using the polymer film as the electrochromic layer and TiO_2_ as the ion storage layer. The electrochromic devices exhibited the maximum absorption at 385 nm and appeared yellow in their initial state (Figure 6). After applying an external potential of 1.9 V, the color of the devices began to darken and turned orange-red, and the absorption band centered at 385 nm slightly weakened, accompanied by a new absorption peak appearing at 485 nm. When the driving voltage was increased to 2.5 V, the appearance of the devices changed quickly to blue, consistent with the decrease in absorption intensities in the range of 350–530 nm and the rapid enhancement of a new broad absorption band centered at 755 nm. The color of the devices could be fully recovered to its original state by applying a potential of −0.1 V (Figure 6). Thus, a reversible electrochromic device that exhibits three stable color states tunable between yellow, orange-red, and blue by adjusting the external voltage (0, 1.9, 2.5 V, respectively) was successfully obtained.

The first step of the electrochromic process involved the oxidation of Fe(II) to Fe(III) ions, resulting in the increase in absorption intensities in the range of 450–580 nm, while the second step was attributed to the formation of radical triphenylamine, giving rise to strong absorption centered at 755 nm. Therefore, the color switching time, cycling stability, and coloration efficiency of the electrochromic device in these two steps of the electrochromic process were further examined.

**Figure 7:**
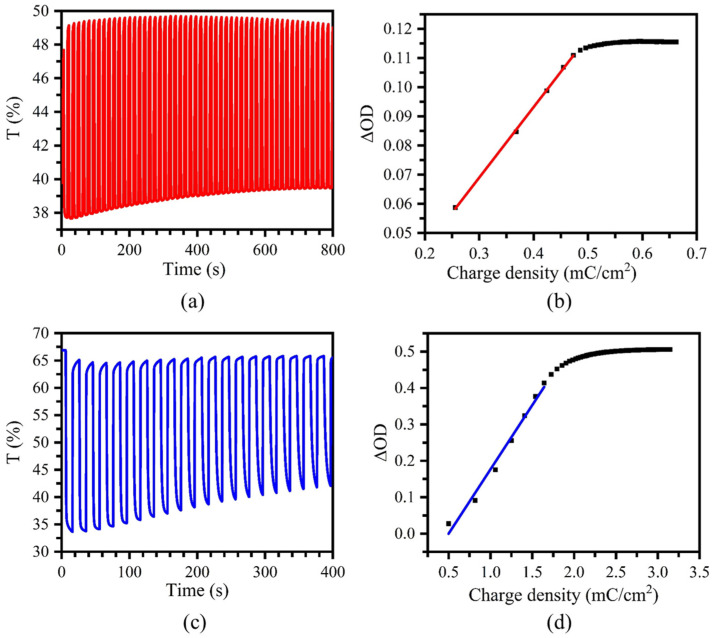
(**a**) Transmittance of the device monitored at 535 nm at the applied voltages of −0.1 and 1.9 V. (**b**) Plot of the optical density (Δ*OD*) versus the charge density (mC/cm^2^) for the device at 1.9 V. (**c**) Transmittance of the device monitored at 755 nm at the applied voltages of −0.1 and 2.5 V. (**d**) Plot of the optical density (Δ*OD*) versus the charge density (mC/cm^2^) for the device at 2.5 V.

With the stepping potentials between –0.1 and 1.9 V at an interval of 10 s, the transmittances at 535 nm of the device were collected, as shown in Figure 7a. The *t*_c_ and *t*_b_ values of the device were calculated to be 1.0 and 1.0 s, which were significantly shorter than those observed in the film measured in the three-electrode system. The optical contrast of the device was found to be Δ*T* = 12%, and a slight drop to 10% after 50 cycles was observed, indicating a good cycling stability of the device when switching between –0.1 and 1.9 V. The coloration efficiency (CE), defined as *η* = ∆*OD*/∆*Q* = (log (*T*_b_/*T*_c_))/∆*Q* (∆OD is the change of the optical density and ∆Q is the quantity of injected electric charges), was calculated to be 241.5 cm^2^/C at 535 nm (Figure 7b).

As shown in Figure 7c, the switching time of the device at the stepping potentials between −0.1 and 2.5 V at 755 nm was estimated to be *t*_c_ = 1.2 s and *t*_b_ = 0.4 s, accompanied by an optical contrast increase to 33%. The CE of the device reached 352.9 cm^2^/C at 755 nm (Figure 7d), with a decrease in optical contrast from 33% to 23% after 20 cycles, which indicated a slightly weaker electrochemical stability of the device switching between −0.1 and 2.5 V.

## 3. Materials and Methods

**Materials and methods.** Complex **Fe1** was prepared according to the literature methods [16]. Other chemicals were purchased from Adamas Reagent Co., Ltd. (Shanghai, China) and used as received unless otherwise stated. Indium tin oxide (ITO)-coated glass slides (20 mm × 100 mm × 1.1 mm, *R*_s_ = 6 Ω/sq) were purchased from South China Xiangcheng Technology Co., Ltd. (Shenzhen China), and cleaned by sequentially washing them with an alkaline detergent, deionized water, ethanol, acetone, and isopropanol before use.

**Instrumentation**. UV–vis absorption spectra were measured on a UV2600i UV–vis spectrophotometer (Shimadzu, Suzhou, China). Fourier-transform infrared (FT-IR) spectra were recorded on a Bruker VERTEX 70 FT-IR spectrophotometer with a Platinum ATR module (Berlin, Germany). Cyclic (CV) voltammograms were recorded with an electrochemical analyzer (CHI 660E, Chenhua, Shanghai, China) in acetonitrile solutions containing 0.1 M (Bu_4_N)PF_6_ as the supporting electrolyte. Platinum, glassy graphite, and Ag/AgCl were used as the counter, working, and reference electrodes, respectively.

**Continuous in situ polymerization of electrochromic films. Fe1** (10.0 mg), di-/tri-aldehyde precursors (**A1**–**A4**, 2.0 equiv.), and chloroform (20 mL) were added to a 50 mL Schlenk tube under an N_2_ atmosphere. An ITO glass slide (20 mm × 100 mm) was immersed into the solution. After the solution was heated up to 50 °C, 0.5 mL of trifluoroacetic acid were added. After standing for 8 h, the ITO glass slide was removed, ultrasonically cleaned with a triethylamine and acetonitrile solution to remove the trifluoroacetic acid and oligomers, and dried in the air.

**Characterization of electrochromic films.** In situ synchronizing spectroelectrochemistry studies were conducted using a combination of an CHI 660E electrochemical workstation and a UV2600i UV–vis spectrometer. The electrochromic films (**P1**–**P4**) were used as the working electrodes to set up the three-electrode system in a quartz cuvette using a platinum plate as the counter electrode and Ag/AgCl as the reference electrode. The cuvette was placed in the sample holder of the UV–vis spectrometer and connected to the electrochemical analyzer. The absorption spectra of the films under different applied voltages were collected simultaneously during the electrochemical measurements.

**Fabrication of electrochromic devices.** Electrochromic devices (ECDs) were fabricated with a sandwich structure of ITO/electrochromic film/gel electrolyte/TiO_2_/ITO. TiO_2_ was chosen as the ion storage layer and prepared using a sol–gel method. The TiO_2_ thin film was deposited on the ITO glass using the pulling technique and annealed at 450 °C. After mixing polymethyl methacrylate (PMMA, 1.75 g), LiCF_3_SO_3_ (0.58 g), acetonitrile (4 mL), and propylene carbonate (PC, 4 mL), the mixture was stirred for 12 h under 70 °C to produce the gel electrolyte. The electrochromic film and the TiO_2_-coated ITO glass were joined together with the gel electrolyte. The device was then annealed at 70 °C for 8 h and used for subsequent characterizations.

**Characterization of electrochromic devices.** The device was directly placed in the sample holder of the UV–vis spectrometer and connected to the electrochemical analyzer using a two-electrode configuration. The absorption or transmittance spectra of the devices under different applied voltages was monitored simultaneously during the electrochemical measurements.

## 4. Conclusions

We developed a continuous in situ polymerization method to prepare an electrochromic film on conductive ITO glass based on the Schiff base condensation between a tetraamine Fe-based complex and an organic di-/tri-aldehyde precursor. The so-formed polymer film uniformly adhered to the surface of ITO even after the treatments of electrochemical CV cycles and/or ultrasonication in a common organic solvent. The thickness of the film was easily tunable by changing the concentration of reaction precursors. Film **P1** originated from **Fe1**, and **A1** underwent two reversible redox processes that present two steps of electrochromic processes. Under a low external potential of 1.05 V, the oxidation of Fe(II) to Fe(III) at 1.05 V led to the color of the film changing from yellow to orange-red. After applying a slightly higher external voltage of 1.4 V, film **P1** turned to blue due to the oxidation of triphenylamine moieties to cation radicals. Electrochromic film **P1** exhibited good electrochemical stability and fast switching times of *t*_c_ = 1.9 s and *t*_b_ = 1.6 s, with an optical contrast of 45% at 745 nm. Most importantly, excellent electrochromic performance with good cycling stability, very short response time of 0.4–1.2 s, and coloration efficiencies of 241.5–352.9 cm^2^/C at 1.9 V (at 535 nm) and 2.5 V (at 755 nm) was achieved with the electrochromic device based on polymer film **P1** with the structure of ITO/electrochromic film/gel electrolyte/TiO_2_/ITO. Our studies demonstrate a strategy for constructing a uniform and stable electrochromic polymer film based on a metal complex precursor, thus providing a feasibility approach to developing high-performance electrochromic devices.

## Data Availability

Dataset available upon request from the authors.

## Supplementary Materials

The following supporting information can be downloaded at: https://www.mdpi.com/article/10.3390/molecules30051099/s1, Figure S1. FT-IR spectra of film **P2** comparing with the monomer precursor; Figure S2. FT-IR spectra of film **P3** comparing with the monomer precursor; Figure S3. FT-IR spectra of film **P4** comparing with the monomer precursor; Figure S4. AFM images of film **P1**; Figure S5. SEM images of film **P2**; Figure S6. AFM images of film **P2**; Figure S7. SEM images of film **P3**; Figure S8. AFM images of film **P3**; Figure S9. SEM images of film **P4**; Figure S10. AFM images of film **P4**; Figure S11. Plots of cyclic voltammograms of film **P2**; Figure S12. Plots of cyclic voltammograms of film **P3**; Figure S13. Plots of cyclic voltammograms of film **P4**; Figure S14. Electrochromic switching times and stability of film **P2**; Figure S15. Electrochromic switching times and stability of film **P3**; Figure S16. Electrochromic switching times and stability of film **P4**.

## Abbreviations

The following abbreviations are used in this manuscript:

ECDs	electrochromic devices
ITO	indium tin oxide
SEM	scanning electron microscopy
EDS	energy-dispersive X-ray spectroscopy
LMCT	ligand-to-metal charge transfer
*t*_c_	coloration time
*t*_b_	bleaching time
CE	coloration efficiency
∆OD	change of the optical density
∆Q	quantity of injected electric charges
