# Supplementary material for: Continuous In-Situ Polymerization of Complex-Based Films for High-Performance Electrochromic Devices
Source: Molecules. 2025 Feb 27;30(5):1099. doi: 10.3390/molecules30051099 (PMC11902238; doi:10.3390/molecules30051099)
Supplement: Supplementary file 1 [file molecules-30-01099-s001.zip › molecules-3474583-supplementary.pdf]

Supporting Information

Continuous In-Situ Polymerization of Complex-Based Films for High Performance Electrochromic Devices

Yang-Bo Liu [1,2], Hao-Tian Deng [1,2], Li-Yi Zhang [2], Jing-Hao Wei [2], Feng-Rong Dai [2,3,*] and Zhong-Ning Chen [1,2,3,*]

[1] College of Chemistry, Fuzhou University, Fuzhou, Fujian 350108, China

[2] State Key Laboratory of Structural Chemistry, Fujian Institute of Research on the Structure of Matter, Chinese Academy of Sciences, Fuzhou 350108, China

[3] Fujian College, University of Chinese Academy of Sciences, Fuzhou 350002, China

* Correspondence: dfr@fjirsm.ac.cn (F.-R.D.); czn@fjirsm.ac.cn (Z.-N.C.)

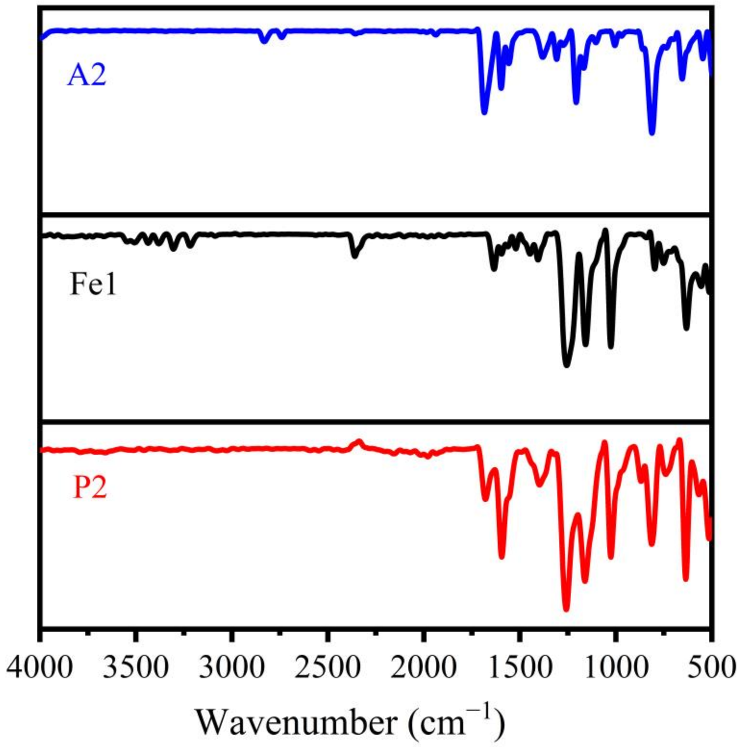

Figure S1. FT-IR spectra of film **P2** comparing with the monomer precursor.

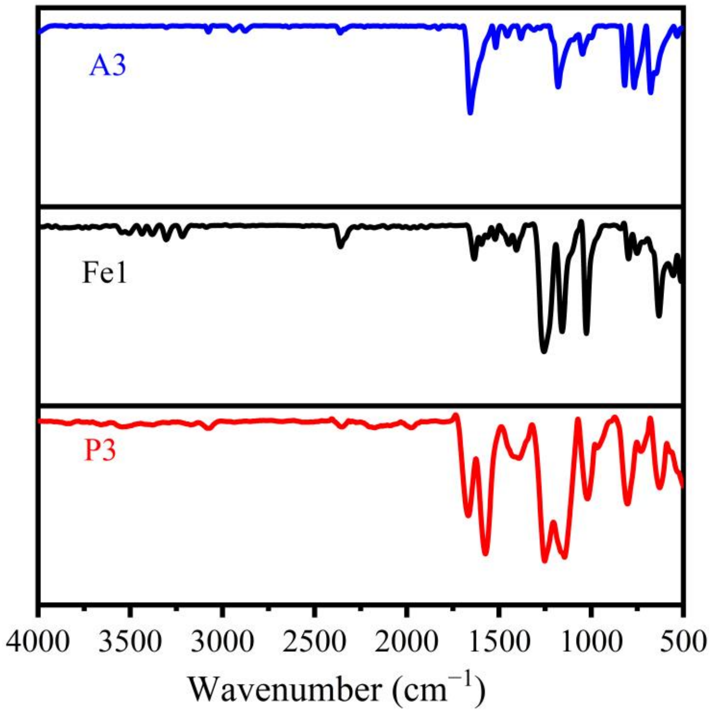

Figure S2. FT-IR spectra of film **P3** comparing with the monomer precursor.

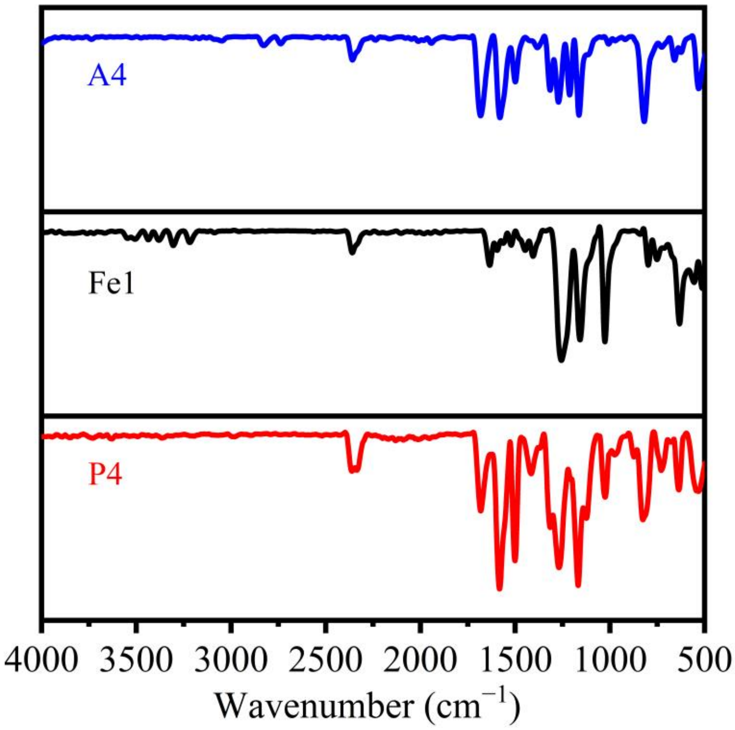

Figure S3. FT-IR spectra of film **P4** comparing with the monomer precursor.

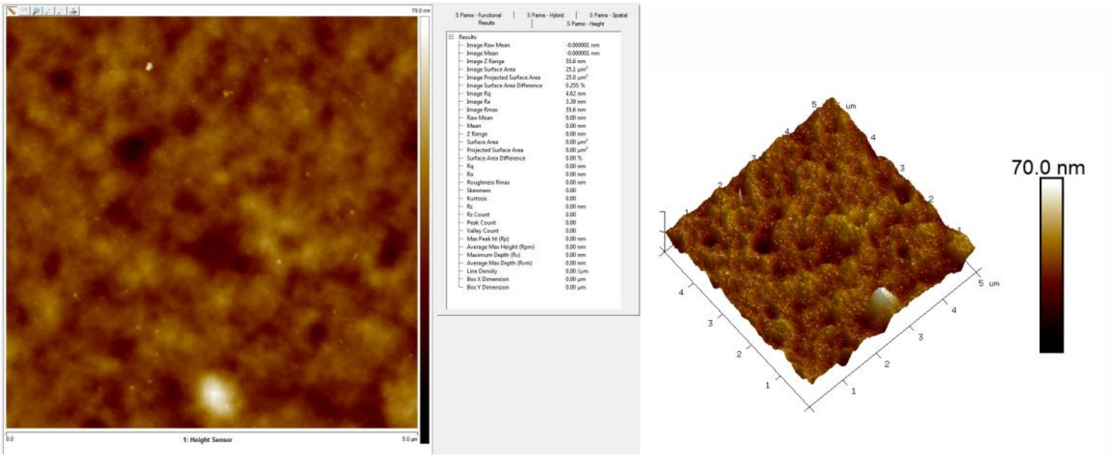

Figure S4. AFM images of film **P1**.

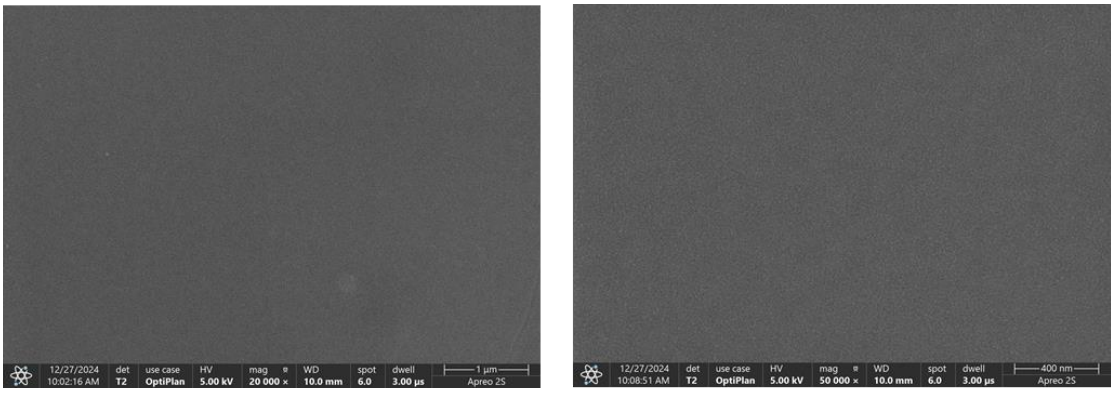

Figure S5. SEM images of film **P2**.

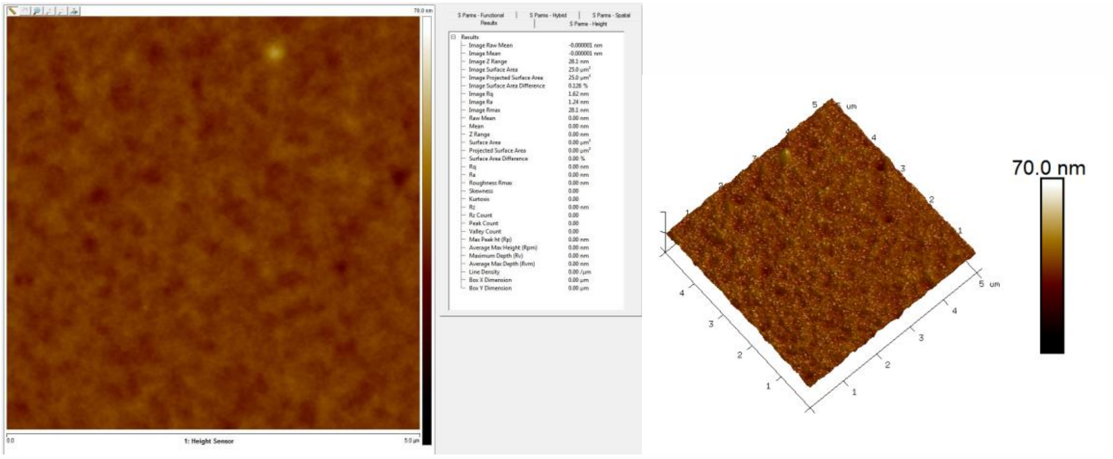

Figure S6. AFM images of film **P2**.

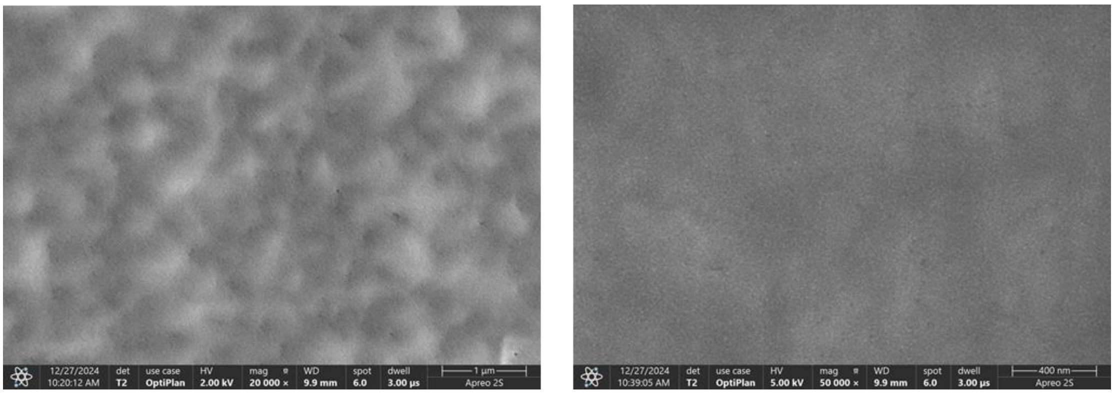

Figure S7. SEM images of film **P3**.

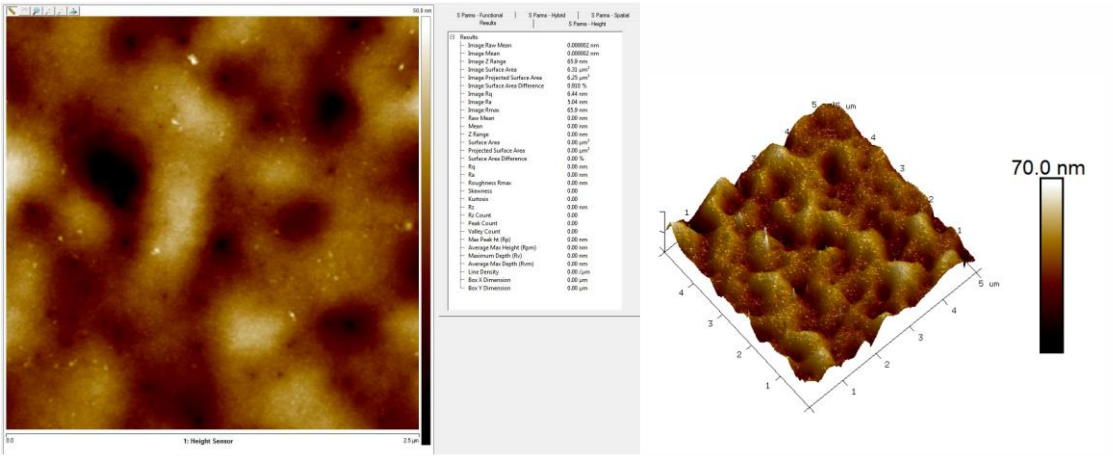

Figure S8. AFM images of film **P3**.

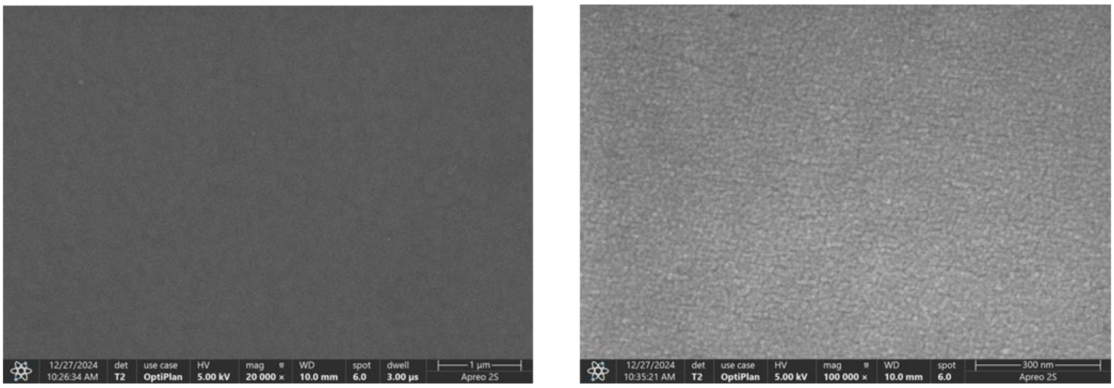

Figure S9. SEM images of film **P4**.

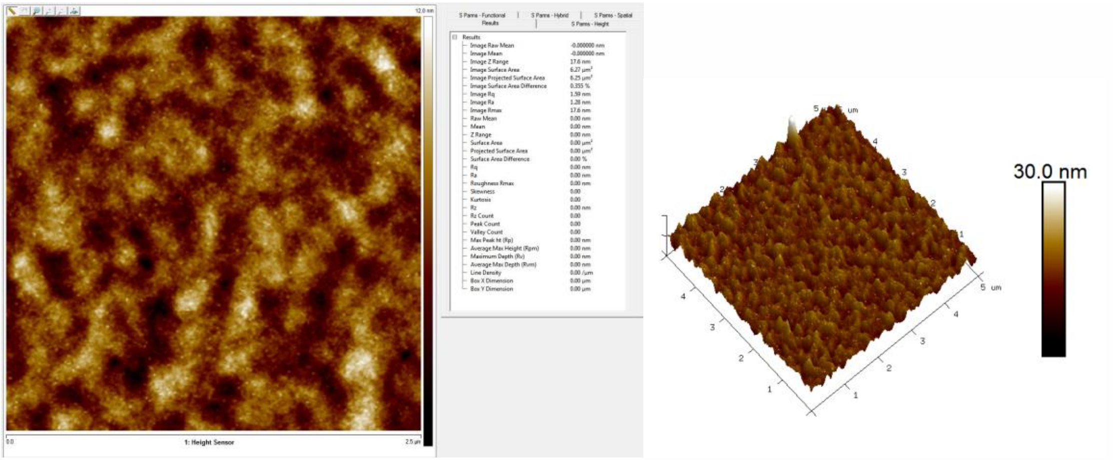

Figure S10. AFM images of film **P4**.

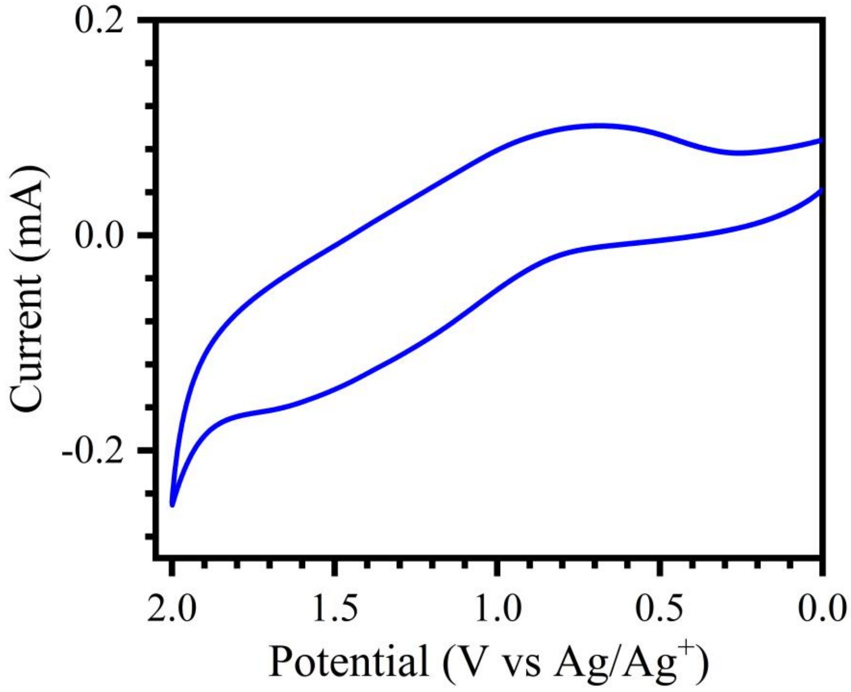

Figure S11. Plots of cyclic voltammograms of film **P2**.

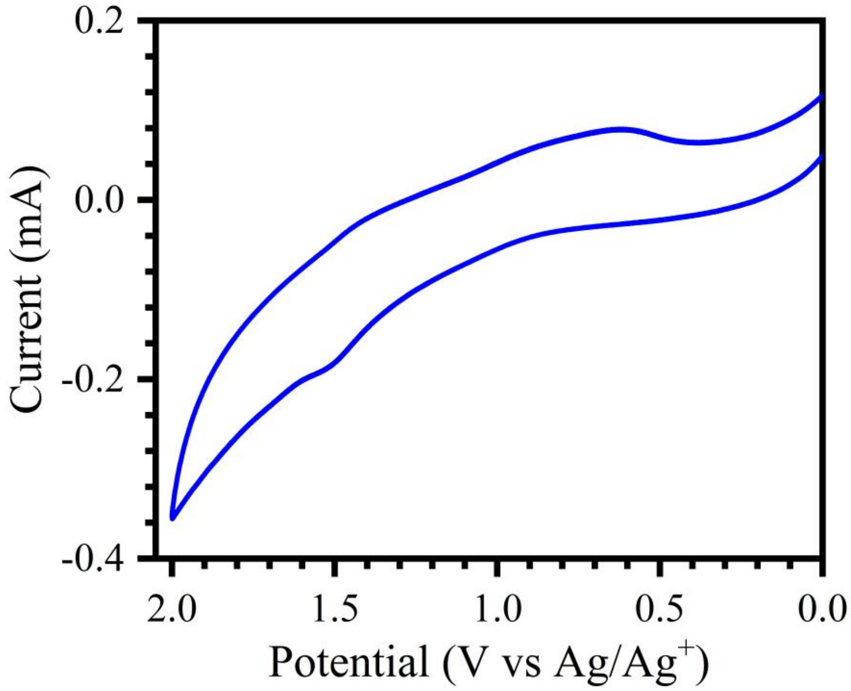

Figure S12. Plots of cyclic voltammograms of film **P3**.

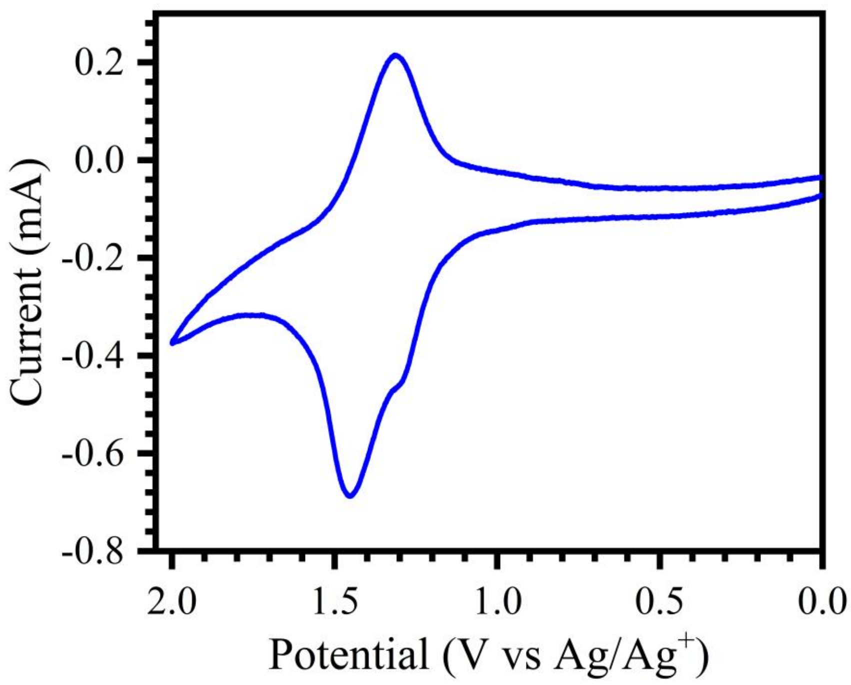

Figure S13. Plots of cyclic voltammograms of film **P4**.

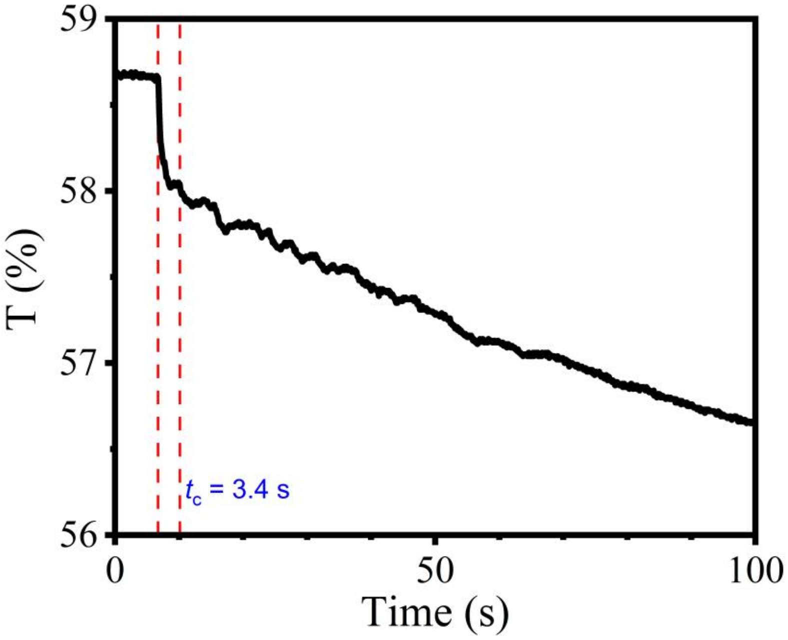

Figure S14. Electrochromic switching times and stability of film **P2**.

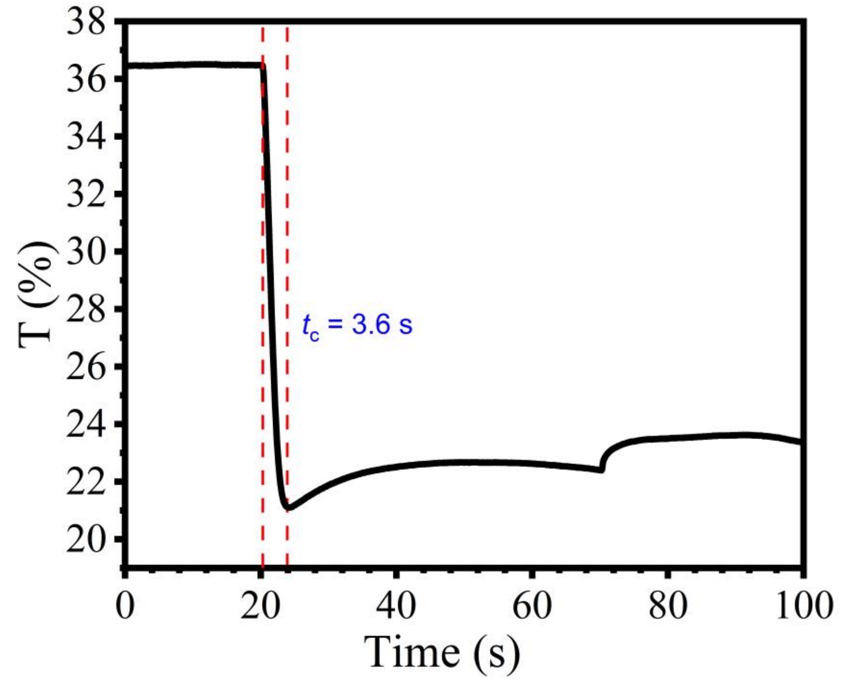

Figure S15. Electrochromic switching times and stability of film **P3**.

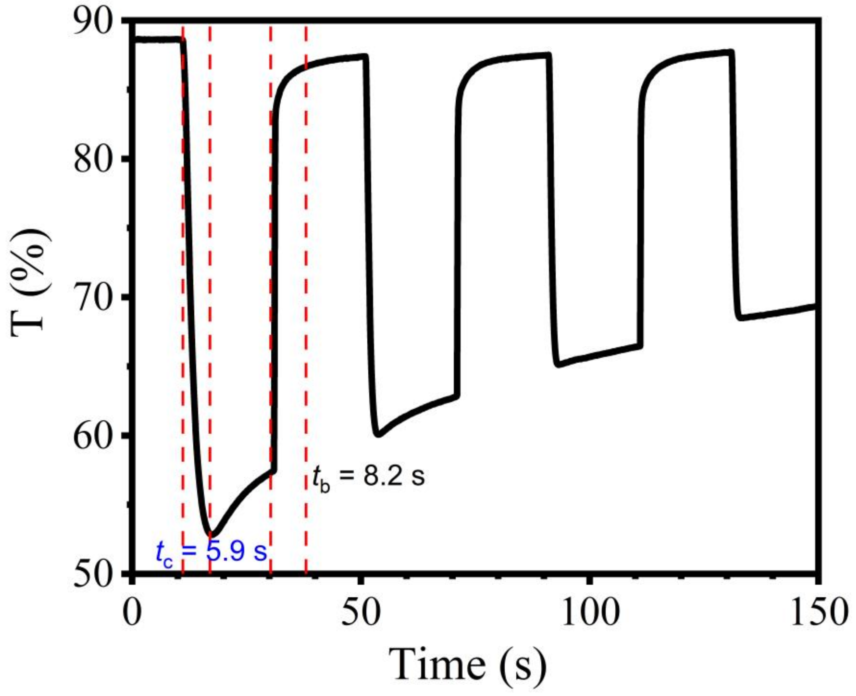

Figure S16. Electrochromic switching times and stability of film **P4**.
